# Perceptions of preparedness: How hospital-based orientation can enhance the transition from academic to clinical learning

**DOI:** 10.36834/cmej.61649

**Published:** 2020-08-06

**Authors:** Lindsay Beavers, Voula Christofilos, Christinne Duclos, Kelly McMillen, Jasmine Sheehan, Laura Tomat, Lianne Jeffs, Rebecca Kelsey, Beverly Bulmer

**Affiliations:** 1 Unity Health Toronto, Ontario, Canada; 2 University of Toronto, Ontario, Canada; 3 North York General Hospital, Ontario, Canada; 4 Sinai Health System, Ontario, Canada; 5 Hospital for Sick Children, Ontario, Canada; 6 University Health Network, Ontario, Canada; 7 St. Joseph's Health Centre, Ontario, Canada; 8 Toronto Academic Health Science Network, Ontario, Canada

## Abstract

**Background:**

Clinical placements are essential for applied learning experiences in health professions education. Unfortunately, there is little consensus on how best to prepare learners for the transition between academic and clinical learning. We explored learners’ perceptions of hospital-based orientation and resulting preparedness for clinical placement.

**Methods:**

Sixty-three learners participated in a total of 18 semi-structured focus groups, during their clinical placements. Data were analyzed thematically.

**Results:**

We organized learners’ perceptions of hospital-based orientation that support their preparedness for placement into three themes: (1) adequate *site* orientation for learner acquisition of organization acumen and (2) clinical preceptor training to support *unit/service* and (3) *individual* components.

**Conclusion:**

Thoughtful attention to hospital-based orientation can support learners in transitioning from academic to clinical learning. Hospital organizations should attend to all three components during orientation to better support learners’ preparedness for clinical learning.

## Abstract (fr)

**Contexte:**

Les stages cliniques sont essentiels aux expériences d’apprentissage appliquées dans l’enseignement médical. Malheureusement, il n’existe pas de consensus sur la meilleure manière de préparer les étudiants à la transition entre l’apprentissage académique et l’apprentissage clinique. Nous avons exploré la perception des étudiants de l’orientation en milieu hospitalier et de l’état de préparation qui en résulte pour les stages cliniques.

**Méthodes:**

Au total, 63 étudiants ont participé ’à travers 18 groupes de discussion semi-structurés lors de leurs stages cliniques. Les données ont été analysées de manière thématique.

**Résultats:**

Nous avons classé les perceptions des étudiants relativement à l’orientation en milieu hospitalier qui soutient leur état de préparation pour un stage en trois thèmes différents : (1) une orientation adéquate du *site* pour l’acquisition par l’étudiant de l’expertise de l’organisation et (2) la formation du précepteur clinique pour soutenir *l’unité/le service* et (3) les composantes *individuelles*.

**Conclusion:**

Une attention particulière centrée sur l’orientation en milieu hospitalier peut soutenir les étudiants dans leur transition de l’apprentissage académique à l’apprentissage clinique. Les organismes hospitaliers doivent inclure les trois composantes pendant l’orientation pour mieux soutenir l’état de préparation des étudiants pour leur apprentissage clinique.

## Introduction

An essential component of health professionals’ training is applying theoretical and conceptual knowledge in the clinical context. Experiential learning during clinical placements offers learners the opportunity to practice and demonstrate the combination of knowledge, attitudes, and skills in complex care environments.^1^ Academic leaders of health professions education programs recognize the importance of these clinical experiences, and often proactively work to support learner preparedness for clinical placements. Adding to the importance of this topic for clinical sites, recent provincial legislative changes to the *Occupational Health and Safety Act*^2^ in Ontario identify learners as ‘workers’, therefore Ontario hospitals have a statutory responsibility in preparing learners for clinical learning.

A key enabler in preparing learners to successfully transition between academic and clinical learning is orientation. Orientation for medical and nursing learners transitioning from the academic settings to clinically-based learning has been explored in the literature.^3^^–^^6^ We wanted to explore orientation activities for learners that occur within the hospital setting, or ‘hospital-based orientation’ (HBO). Newly graduated staff nurse orientation has been extensively explored,^7^^,^^8^ however, we were unable to find any literature exploring learners’ perceptions of HBO.

In 1998 a team of Australian medical researchers created a *Preparedness for Hospital Practice* survey tool in order to evaluate the adequacy of junior doctor’s undergraduate medical training in preparing them for hospital practice.^9^ The survey defined eight domains of preparedness for clinical learning that focused on learners’ self-reported skill and knowledge acquisition.^9^ Many academic institutions also focus on skills and knowledge improvements during the transition from academic to clinical learning, using multiple strategies and approaches.^10^^-^^14^

Interestingly, and perhaps in slight contrast to the training as orientation mentioned above, rehabilitation sciences preceptors and learners (occupational therapy, physiotherapy, and speech-language pathology) identify the learners’ personal attributes and interpersonal skills - such as willingness, professionalism, communication and interaction, and personal attributes and skills, as key indicators of preparedness for clinical learning.^15^^,^^16^

In a study of recent nursing graduates, medical learners, and organizational representatives, four dimensions of work readiness (‘the extent to which graduates possess the attributes that prepare them for success in the workplace’) were identified: *work competence, personal characteristics, social intelligence*, and *organizational acumen*.^17^ For us, the organizational acumen dimension in particular could be a useful lens to understand how HBO prepares learners to successfully transition from academic to clinical learning.

As a first step towards supporting learners transitioning from academic to clinical learning, we sought to understand the learner perception of HBO. We asked: 1) how do learners perceive HBO?, and 2) how does HBO impact clinical learners’ perceptions of preparedness?

## Methods

We used a qualitative description methodology to explore learner perceptions of HBO and preparedness across nine hospital sites across the Greater Toronto Area.^18^ The Research Ethics Boards at all participating hospitals approved this study (Toronto Academic Health Science Network Research Ethics Board #16-380).

### Participants

We used convenience sampling to recruit learners who were on placement of at least two weeks from February-October, 2017. Recruitment across all hospital sites involved distributing electronic and paper flyers. Learners self-selected to participate in the study and received no remuneration.

A total of 63 learners agreed to participate [51% (*n* = 32) from nursing, 11% (*n* = 7) from medicine and 38% (*n* = 24) from allied/health disciplines (physiotherapy, occupational therapy, respiratory therapy etc.)]. All learners participated in a focus group at the site where they were attending placement. As we were exploring learners’ perceptions of what orientation was, we did not select a target window between the start of a learner’s placement and participation in the study.

### Data collection and analysis

The lead author or the research assistant led semi-structured focus groups. We conducted eighteen, 45-60-minute focus groups. Sixteen consisted of two to ten participants per site, and two of the planned focus groups became individual interviews due to participant attrition. Learners self-selected focus group dates, resulting in seven of the focus groups being uniprofessional, and the mixed. We recorded and transcribed verbatim the focus groups. The lead author and a research assistant carefully read and coded all transcripts, and held meetings monthly to distill initial themes and modify the interview guide. The core research team (LB, VC, RK, LJ, and BB) analyzed the entire data set with a final coding structure. We stopped collecting data once no new information or themes were being identified.^19^^,^^20^

## Results

Orientation consisted of three themes organized by three components: *site, unit/service, and individual*. These three components influenced learners’ perception of preparedness for their clinical placement. See Table 1. for representative quotations of each.

**Table 1:** Learners’ perceptions of orientation

Orientation	Learner quotes
	Hospital
**Hospital specific knowledge**	*‘…every member in the hospital is responsible for taking part in this… it is just kind of reassuring… that there are procedures to set up in place and this is what to do, you’re not alone, that sort of encouragement.’* (Focus Group #12) *‘…it [orientation] just gives you a bigger picture as opposed to only focusing on your floor, and I just feel like if everybody is involved*.’ (Focus Group #3)
**Access to systems**	*‘We already got our PowerChart logins and everything, so on our first day on the unit it never took away time to do that stuff so we got to spend our time on the unit.’* (Focus Group # 1) *‘We had to do an online module and then do in-person orientation training for it [electronic medical record access], and that was important because then we could access information about the people we were seeing’* (Focus Group # 6)
**Learning efficiency**	*‘With a good orientation, definitely you also save a lot of time. If you don’t know where certain places are, then you are wasting your time that you could be using for learning and other opportunities.’* (Focus Group # 18) *‘it [hospital site] was very confusing, so because it’s very spread out and it’s very large, it was a little overwhelming at first, but I felt we had a good orientation in the beginning…just to make sure we understood where to go’ (*Focus Group #4)
	**Unit/service**
**Unit/service specific knowledge**	*‘…even though I’ve passed by it a million times, I still have the orienting. Which means, where do I look for certain things…what’s the workflow like? Who do I report to in case I have questions? Who do I get in touch with? What happens in case of a mistake?’* (Focus Group #12) *‘…knowing the resources that are available on the floor is really nice. We weren’t aware that there’s only two vital signs machines on our unit that work, for I don’t know how many beds our unit is, but it’s a lot of beds to split two vital signs machines for. Just knowing exactly how much you’re going to fight for resources, and where to find things…’ (*Focus Group # 10)
**Safety**	*‘Yeah, for the wards that are a bit … psych and also like found in the Emergency Room … what to do if you know a patient is aggressive or you’re uncomfortable it’s like how to extract yourself. I don’t know if you’d need that at every rotation.’* (Focus Group #2) *‘Safety, would be a big thing, especially on the mental health inpatient unit. As I worked there, I heard more stories and I was like, oh, yeah, this place can be kind of dangerous...’* (Focus Group #5)
**Collaboration**	‘*Being introduced to everyone makes a really big difference in how comfortable you feel in that role…everyone knows who you are so you don’t feel like you’re just some student in the corner, you don’t have to introduce yourself. And you know who to approach…you have that independence because you are familiar with the environment you’re working in.’* (Focus Group #7) *‘I’ve kind of got to see, and ask the question of, well, how do you work with recreational therapy, just to orient myself with that patient population, and how we collaborate together which is great.’* (Focus Group #9)
**Escalation of care**	*‘I met with the staff…we had a quick discussion of what were anticipations and expectations, and how do we manage problems on the floor…so there are clear things that were put forward before we even started….so if I was ever worried about managing a case, I knew I had help or back up there.’* (Focus Group #18) *‘…it’s [orientation] like the gateway to understand a lot of logistical stuff like the policies, but also to have some ideas if there’s any problems to troubleshoot moving forward, who would you contact…’* (Focus Group # 17)
	**Individual**
**Matching goals**	*‘It’s just helpful to know kind of what’s what and what you’re expected to do [as per the preceptor] and then you can make your own goals around that as well.’* (Focus Group #2) *She [preceptor] is so supportive, and she even takes into account the background that I do have…She does take that into consideration which is very … I really appreciate it. Even I’ve noticed some of the other professionals, as soon as they find out that I’m also that [occupational therapy/physiotherapy assistant], not that they value me more, but they take that into account when they’re speaking to me…’* (Focus Group #11)
**Roles and responsibilities**	*‘[orientation]is intended to let residents know what their roles and responsibilities are for whatever teaching they are getting at whatever site… orientating them to the guidelines or other kinds of component service that may be necessary for their jobs.’* (Focus Group # 16) *‘For an orientation, I value that. The preceptor has an understanding of what their own role is, for sure, not just what the student or the preceptee is meant to bring.’* (Focus Group #1)
**Preceptor expectations**	*‘But, I think it really depends on what you’re doing here. For example, if a person has multiple preceptors, preceptors have different expectations in terms of how to operate in certain units. And so there will be specific unit/department orientations, if you want to call it that.’* (Focus Group #12) *‘[Preceptor]…laid out the expectations for us. I think that’s something that’s really important, because coming in as a student, you really want to know how you can succeed in the rotation…, and how to gauge at each level of our rotation where we should be, which was really nice.’* (Focus Group #15)

### Site

The first component of orientation, *site*, consisted of organizational requirements learners needed to complete to move forward with their placement. Learners described gaining site-specific knowledge that supported their perception of inclusion in the clinical setting.

This component also included gaining access to electronic systems, receiving security identification badges, and information about where to go on the first day. Learners also appreciated that orientation had the ability to increase the efficiency with which they could begin learning.

### Unit/service

The second component of orientation, *unit/service*, included knowledge or training that learners perceived enabled them to learn effectively and safely in that specific unit/service area and with that team. Learners identified the need for area-specific information such as tours of areas, schedules for training, and where items are located. Learners also identified that the orientation had to be specific to each clinical area, as tasks and processes were different in each setting. Knowledge gained in this component facilitated learners’ perceptions of their ability to safely function in that specific area/unit. Learners’ perception of the completeness of their unit/service orientation also impacted their perceived ability to collaborate with the team. Finally, learners identified knowledge and insights about the members of the team, and their roles as a way to prepare to respond to situations as they arose.

### Individual

The third component of orientation was *individual*. Learners described where they were in their academic journey, including an understanding of their learning goals, roles, responsibilities, and accountabilities. Discussions with their preceptors significantly shaped this understanding. Learners also perceived that having clearly defined responsibilities supported them to identify their own roles within the team. Learners identified how to be successful in their placement, through clear preceptor expectations.

## Discussion

A health professions learner is successful when they are able to consistently demonstrate a certain level of competence, appropriate for their level of training, and required for their profession. Clinical placements contribute substantially to this process. Our study provides some insight into learners’ preparedness to transition from academic to clinical learning by identifying three major components of HBO (*site, unit/service & individual)*. Work readiness dimensions of work competence (knowledge and skills)^11^^-^^14^^,^^15^^,^^21^^-^^23^ and personal characteristics^15,^^16^ have been explored in studies, however, we could not find studies detailing the organizational acumen dimension (knowledge of the area, procedures, and policies) and its relationship to learner preparedness. Our findings suggest that opportunities to develop specific organizational acumen during HBO could play an important factor in helping learners feel prepared transitioning between academic and clinical learning. Our results demonstrate that learners are able to consistently articulate what HBO is and how it impacts their preparedness, despite participants representing multiple professions, and experiencing different HBO’s at different times.

Learners were seeking out tangible knowledge and items that facilitated access to learning opportunities within the clinical setting in the s *ite* component of orientation. Learners found it extremely difficult, if not impossible to fully participate in the clinical setting without access to the clinical site’s policies and procedures, electronic systems etc. (organizational acumen). These findings are consistent with the categories within Walker et al.’s work readiness study, which indicated the need for specific knowledge related to the clinical sites’ policies and procedures.^17^

Learners clearly identified the requirements of being prepared to learn, including orientation items that promoted learner safety in the *unit/service* component of orientation. This included understanding of the learner specific role in difficult situations, and/or identification of key support persons to ask for assistance when learners felt unsafe. Amy Edmonson’s work related to psychological safety in teams reinforces these findings.^24^^-^^26^ Edmonson defines psychological safety as ‘the degree to which people perceive their work environment as conducive to taking these interpersonal risks’^24^ and argues that in psychologically safe environments, learning is fostered by the individuals’ belief that they are safe to seek assistance and support.^26^

Finally, learners benefited from having clearly identified roles and expectations for success. This meant specific opportunities to perform the skills and tasks of their profession in the *individual* component of orientation. A clinical preceptors’ ability during HBO to support learners’ preparedness is reflected in the literature through best teaching practices. Sutkin’s 2008 scoping review of what makes a good clinical teacher identified teacher characteristics such as ‘organized and communicates objectives’ and ‘individualized attention to students’ and ‘stimulates or inspires trainees thinking’ which aligns with the learners’ perceptions in this study.^27^

**Table 2:** Components of orientation and key requirements for learner preparedness.

Components of Orientation	Key requirements for learners’ preparedness
Site	Timely completion of placement prerequisites (e.g. immunization records, mask fit testing, and completion of on-line safety and training modules) Acquisition of tangible items (e.g. security identification badges, scrubs, pagers, lockers and access to electronic systems) Knowledge of safety processes and procedures (e.g. infection control processes, or how to call a code)
Unit/service	Tours and knowledge of unit/service specific resources Personal introductions to other team members Identification of person(s) to assist the learner in unsafe situations Identification of roles, both the of the learner and role of the team members
Individual	Discussion and attainment of specific learning objectives with preceptor Support and opportunity for skill development by preceptor and team

The results of this study provide the opportunity to conceptualize learners’ preparedness for successfully transitioning between academic and clinical learning, as the ‘ability to safely learn and perform in the clinical environment through the acquisition of organization-specific knowledge and access, socialization into their unit/service area and identification of expectations and goals.’

As hospitals have an obligation to support clinical learners, we wish to highlight two key elements over which hospitals have a degree of control during HBO planning and resource allocation. The first is providing adequate HBO that supports learners’ acquisition of organization acumen. The second is ensuring clinical preceptors have the training required to support components of orientation in which they play a large role, i.e. the *unit/service* and *individual* components. In conclusion, as hospitals and academic institutions continue to work closely together to prepare learners, to identify roles and responsibilities, and to provide clear systems of communication will be key in ensuring consistent messaging and adequate systems of support for learners.

## Limitations

The different compositions of our focus groups (intraprofessional vs. interprofessional) could have created different dynamics in each group, thus potentially impacting the content of the discussions. Participants also had different orientation timelines and experiences depending on their program of study and specific hospital placement. While the differences facilitated an exploratory approach to the topic, it also limits our ability to narrow findings to a specific learner group or generalize beyond our own system.

## Conclusion

This study provides preliminary information related to the relationship between HBO and learners’ perceptions of preparedness for transitioning between academic and clinical learning. Currently, academic institutions’ focus on preparing learners includes an emphasis on knowledge and skill acquisition and we argue HBO can also play an integral role in learners’ preparedness at the *unit/service* and *individual* levels. First, HBO ensures that learners obtain the site’s organizational acumen, and second, HBO prepares clinical preceptors for their role through basic faculty development programming.

Although we explored learners’ preparedness for clinical placement with HOB, we did not explore the relationship between academic institutions compared to HBOs. Health profession group-specific explorations of orientation could be an important next step in determining different learner groups’ perceptions as well as the relationship between academic and HBO.

## References

[1] Chan D Development of the clinical learning environment inventory: using the theoretical framework of learning environment studies to assess nursing students' perceptions of the hospital as a learning environment. J Nurs Educ. 2002;41(2):69-75. 10.3928/0148-4834-20020201-06 11852986

[2] Ontario Provincial Government. Occupational Health and Safety Act, R.S.O. 1990, c. O.1.

[3] Ellaway RH, Cooper G, Al-Idrissi T, Dubé T, Graves L Discourses of student orientation to medical education programs. Med Educ Online. 2014;19 10.3402/meo.v19.23714 PMC3957739 24646440

[4] Zimmerman PA, Eaton R, van de Mortel T Beyond orientation: Evaluation of student lifecycle activities for first-year Bachelor of Nursing students. Collegian. 2017; 10.1016/j.colegn.2017.02.004

[5] Poncelet A, O'Brien B Preparing medical students for clerkships: A descriptive analysis of transition courses. Acad Med. 2008;83(5):444-51. 10.1097/ACM.0b013e31816be675 18448897

[6] Fernandez GL, Page DW, Coe NP, Lee PC, Patterson LA, Skylizard L, et al Boot camp: Educational outcomes after 4 successive years of preparatory simulation-based training at onset of internship. J Surg Educ. 2012;69(2):242-8. 10.1016/j.jsurg.2011.08.007 22365874

[7] van Rooyen DRM, Jordan PJ, ten Ham-Baloyi W, Caka EM A comprehensive literature review of guidelines facilitating transition of newly graduated nurses to professional nurses. Nurse Educ Pract. 2018;30(February):35-41. 10.1016/j.nepr.2018.02.010 29524807

[8] Connelly LM, Hoffart N A research based model of nursing orientation. J Nurs Staff Dev. 1998;14(1):31-9. 9661404

[9] Hill J, Rolfe IE, Pearson SA, Heathcote A Do junior doctors feel they are prepared for hospital practice? A study of graduates from traditional and non-traditional medical schools. Med Educ. 1998;32(1):19-24. 10.1046/j.1365-2923.1998.00152.x 9624395

[10] Blackmore C, Austin J, Lopushinsky S, Donnon T Effects of postgraduate medical education 'boot camps' on clinical skills, knowledge, and confidence. J Grad Med Educ. 2014;(December):643-52. 10.4300/JGME-D-13-00373.1 26140112 PMC4477555

[11] Dearmon V, Graves RJ, Hayden S, Mulekar MS, Lawrence SM, Jones L, et al Effectiveness of simulation-based orientation of baccalaureate nursing students preparing for their first clinical experience. J Nurs Educ. 2012;52(1):29-38. 10.3928/01484834-20121212-02 23230885

[12] McNamara N Preparing students for clinical placements: The student's perspective. Nurse Educ Pract. 2015;15(3):196-202. 10.1016/j.nepr.2014.11.011 25586339

[13] Dalwood N, Maloney S, Cox N, Morgan P Preparing Physiotherapy students for clinical placement: student perceptions of low-cost peer simulation. A mixed-methods study. Simul Healthc. 2018;13(3):181-7. 10.1097/SIH.0000000000000276 29346226

[14] Farahat E, Rice G, Daher N, Heine N, Schneider L, Connell B Objective structured clinical examination (OSCE) improves perceived readiness for clinical placement in nutrition and dietetic students. J Allied Health. 2015;44(4):208-14. 26661699

[15] Chipchase LS, Buttrum PJ, Dunwoodie R, Hill AE, Mandrusiak A, Moran M Characteristics of student preparedness for clinical learning: Clinical educator perspectives using the Delphi approach. BMC Med Educ. 2012;12(1). 10.1186/1472-6920-12-112 PMC3527360 23145840

[16] Rindflesch A, Hoversten K, Patterson B, Thomas L, Dunfee H Students' description of factors contributing to a meaningful clinical experience in entry-level physical therapist professional education. Work. 2013;44(3):264-74. 10.3233/WOR-121503 23324678

[17] Walker A, Yong M, Costa B, Fullarton C, Dunning AMT Work readiness of graduate health professionals. Nurse Educ Today. 2012;33(2):116-22. 10.1016/j.nedt.2012.01.007 22336479

[18] Sandelowski M Whatever happened to qualitative description. Res Nurs Health. 2000;23:334-40. 10.1002/1098-240X(200008)23:4<334::AID-NUR9>3.0.CO;2-G 10940958

[19] Braun Virginia, Clarke V Using thematic analysis in psychology. Qual Res Psychol. 2006;3(2):77-101. 10.1191/1478088706qp063oa

[20] Saunders B, Sim J, Kingstone T, Baker S, Waterfield J, Bartlam B, et al Saturation in qualitative research: exploring its conceptualization and operationalization. Qual Quant. 2018;52(4):1893-907. 10.1007/s11135-017-0574-8 29937585 PMC5993836

[21] Bojani K, Schears GJ, Schroeder DR, Jenkins SM, Warner DO, Sprung J Survey of self-assessed preparedness for clinical practice in one Croatian medical school. BMC Res Notes. 2009;2:1-6. 10.1186/1756-0500-2-152 19635136 PMC2723124

[22] Hickson H, Williams B, O'Meara P Paramedicine students' perception of preparedness for clinical placement in Australia and New Zealand. BMC Med Educ. 2015;15(1):1-7. 10.1186/s12909-015-0446-7 26438130 PMC4593214

[23] Moore A, Canaway R, O'Brien KA Chinese medicine students' preparedness for clinical practice: an australian survey. J Altern Complement Med. 2010;16(7):733-43. 10.1089/acm.2009.0244 20590483

[24] Edmondson A Psychological safety and learning behavior in work teams. Adm Sci Q [Internet]. 1999;44(2):350-83. 10.2307/2666999

[25] Edmondson AC Managing the risk of learning: Psychological safety in work teams. Harv Bus Rev. 2002;1-38.

[26] Edmondson AC Teamwork in the netcentric organization [Internet]. West, Michael A, Tjosvold Dean, Smith KG editor. International Handbook of Organizational Teamwork and Cooperative Working. West Sussex: John Wiley & Sons Ltd.; 2008 255-276 p. Available from: http://library.uniteddiversity.coop/Money_and_Economics/Cooperatives/%20International_Handbook_of_Organizational_Teamwork_and_Cooperative_Working.pdf [Accessed April 3, 2020].

[27] Sutkin G, Wagner E, Harris I, Schiffer R What Makes a Good Clinical Teacher in Medicine? Acad Med. 2008;83(5):452-66. 10.1097/ACM.0b013e31816bee61 18448899

